# Beyond glycolysis: multifunctional roles of glyceraldehyde-3-phosphate dehydrogenases in plants

**DOI:** 10.1093/hr/uhaf070

**Published:** 2025-03-03

**Authors:** Yunpeng Cao, Jiayi Hong, Han Wang, Mengfei Lin, Yongping Cai, Liao Liao, Xiaoxu Li, Yuepeng Han

**Affiliations:** State Key Laboratory of Plant Diversity and Specialty Crops, Wuhan Botanical Garden, Chinese Academy of Sciences, Wuhan 430074, China; College of Life Sciences, Anhui Agricultural University, Hefei 230036, China; Institute of Horticulture, Anhui Academy of Agricultural Sciences, Hefei 230000, China; Jiangxi Provincial Key Laboratory of Plantation and High Valued Utilization of Specialty Fruit Tree and Tea, Institute of Biological Resources, Jiangxi Academy of Sciences, Nanchang 330224 Jiangxi, China; College of Life Sciences, Anhui Agricultural University, Hefei 230036, China; State Key Laboratory of Plant Diversity and Specialty Crops, Wuhan Botanical Garden, Chinese Academy of Sciences, Wuhan 430074, China; Beijing Life Science Academy, Beijing 102209, China; Tobacco Chemistry Research Institute of Technology Center, China Tobacco Hunan Industrial Co., Ltd., Changsha, China; State Key Laboratory of Plant Diversity and Specialty Crops, Wuhan Botanical Garden, Chinese Academy of Sciences, Wuhan 430074, China

## Abstract

Glyceraldehyde-3-phosphate dehydrogenase (GAPDH), a highly conserved enzyme in the glycolytic pathway, also acts as a moonlighting protein, performing various functions beyond its classical role in glycolysis, such as regulating gene expression, participating in cell signal transduction, and responding to environmental stress. By interacting with various signaling molecules, GAPDH plays a regulatory role in hormone signaling pathways, influencing plant growth and development. Functional plasticity in GAPDH is modulated mainly through redox-driven post-translational modifications, which alter the enzyme’s catalytic activity and influence its subcellular distribution. This review explores the diverse functionalities of GAPDHs in plants, highlighting their significance in plant metabolic processes and stress adaptation.

## Introduction

Over the past two decades, glyceraldehyde-3-phosphate dehydrogenase (GAPDH), traditionally recognized as a key enzyme in the glycolysis pathway, has been identified as a ‘moonlighting’ protein with multiple roles beyond its metabolic function [1–3]. As an enzyme catalyzing the conversion of glyceraldehyde-3-phosphate to 1,3-bisphosphoglycerate, GAPDH was long considered a ‘housekeeping’ protein essential for basic energy metabolism [3, 4]. However, increasing evidence suggests that GAPDH not only contributes to energy production but also engages in diverse cellular processes, including DNA repair, tRNA export, membrane fusion, cytoskeletal dynamics, and programmed cell death [3, 5–7]. These discoveries have profoundly altered the understanding of GAPDH, revealing its critical functions beyond cellular metabolism. The functional versatility of GAPDH, governed by its dynamic subcellular localization and a spectrum of reversible and irreversible post-translational modifications, intricately regulates its catalytic activity and broad involvement in diverse cellular processes [4, 7, 8]. Research has shown that post-translational modifications, including phosphorylation, acetylation, and redox modifications, dictate whether GAPDH participates in processes such as DNA repair, RNA metabolism, or programmed cell death [2, 4]. Additionally, GAPDH’s functional adaptability across subcellular regions, including the cytoplasm, vesicles, mitochondria, and nucleus, is finely tuned by the cell’s metabolic state and external stimuli, underscoring complex regulatory mechanisms that empower GAPDH to respond dynamically to cellular stress and environmental challenges [9]. In plants, GAPDH fulfills complex regulatory roles through multiple isozymes encoded by distinct genes and localized across subcellular compartments, including the cytoplasm, chloroplasts, and mitochondria, where they contribute not only to energy metabolism but also to critical pathways such as photosynthesis, respiration, and carbon metabolism [7, 9, 10]. Notably, plant GAPDH isozymes include the cytoplasmic glycolytic GAPDH and the chloroplast-localized GAPDH involved in the Calvin cycle, each exhibiting distinct functions based on their binding to specific cofactors, such as NAD^+^ or NADP^+2,11^. Moreover, GAPDH in plants plays a crucial role in regulating stress responses, with its activity modulated by post-translational modifications, particularly in response to oxidative stress and environmental factors, similar to its regulatory mechanisms in animal cells [11, 12]. For instance, oxidative modifications can alter the structure of GAPDH, transforming it from a metabolic enzyme into a regulatory protein that interacts with other cellular pathways. This functional shift enables plants to adapt to adverse conditions, such as drought, salinity, and low temperatures, ensuring cellular survival and metabolic homeostasis. In summary, recent research has demonstrated that GAPDH is a dynamic, multifunctional protein with roles in plant cells that extend far beyond its glycolytic function. This review will systematically examine the multifunctionality of GAPDH in plant cells, with a focus on its significance in plant stress responses, metabolic regulation, and development, while also highlighting future research directions on GAPDH’s functions and regulatory mechanisms.

## Diversity and structure of the GAPDHs in plants

Plants exhibit multiple GAPDH isoforms, including cytosolic GAPDH (GAPC) and plastidial GAPDH (GAPA and GAPB), each specialized for unique functions [2, 5]. Plastidial GAPDH isoforms in plants, specifically the chloroplast-localized GAPA and GAPB, are integral to the Calvin cycle and redox regulation, supporting carbon fixation and metabolic adaptation under varying light conditions [13–15]. These enzymes work in concert with the photosynthetic machinery to optimize energy use and carbon assimilation, which is crucial for plant survival under fluctuating light. Unlike cytosolic GAPC, which is NAD-dependent and functions in glycolysis, chloroplast GAPDH isoforms primarily use NADP, aligning with the Calvin cycle’s requirement for NADPH to drive reactions that regenerate and fix carbon during photosynthesis. Through compartmentalization, where cytosolic GAPDH drives glycolysis and plastidial GAPDHs orchestrate the Calvin–Benson cycle, GAPDH exemplifies structural diversity and evolutionary specialization, enabling it to support both energy production and biosynthetic pathways across diverse metabolic functions essential for plant life [16–18].

**Figure 1:**
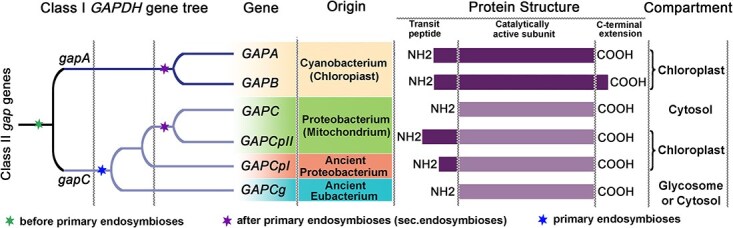
Diagrammatic illustration of the structural features, cosubstrate specificities, cellular localization, and evolutionary origins of eukaryotic GAPDH enzymes and their encoding genes. The diagram is adapted from [19, 20]. The ‘Protein Structure’ highlights the presequence regions of GAPA, GAPB, GAPCpI, and GAPCplI polypeptides, along with the distinctive carboxy-terminal extension present in GAPB, as described by [19, 20]. The ‘Catalytically Active Subunit’ emphasizes their evolutionary derivation from bacterial genes *gapA* and *gapC*, respectively. Notably, four nuclear-encoded *GAPC* gene variants are identified: two (*GAPCg* and *GAPCpI*) appear to have emerged through endosymbiotic gene transfer or lineage separation events predating cryptic endosymbiotic processes, while the other two variants (*GAPC* and *GAPCpII*) likely arose from a gene duplication event within the green algal lineage.

The evolutionary history of GAPDH in plants involves significant gene duplication events [19, 20]. There are six major GAPDH polypeptides found in eukaryotes, arising from distinct lineages: four (GAPA, GAPB, GAPC, and GAPCp) emerged through gene duplications, both before (GAPA/GAPC) and after (GAPA/GAPB, GAPC/GAPCp) the endosymbiotic origin of chloroplasts and mitochondria (Fig. 1). The remaining GAPDH forms may have originated from endosymbiotic gene transfers from ancient eubacteria and proteobacteria or from earlier bacterial lineages, highlighting the complexity of GAPDH evolution in the context of plant endosymbiosis. Notably, GAPDH transit peptides for chloroplast import evolved independently three times: once for GAPA and GAPB, and twice for GAPC, resulting in GAPCpI and GAPCpII forms, which are thought to have anabolic and catabolic functions, respectively [19, 20]. These molecular adaptations provide insight into the evolutionary origins of photosynthesis and the functional diversification of GAPDH in response to metabolic demands [16].

The first functional verification of GAPDH in a plant species was achieved with the chloroplast-localized GAPDH involved in the Calvin–Benson cycle [21, 22]. In recent years, the increasing availability of sequenced plant genomes has allowed for comprehensive genome-wide analyses of the *GAPDH* gene family across a wide range of plant species. Studies have characterized GAPDHs in diverse plants, including *Arabidopsis thaliana*, *Oryza sativa*, *Triticum aestivum*, *Nicotiana tabacum*, *Solanum tuberosum*, *Citrus sinensis*, *Fragaria ananassa*, and *Manihot esculenta*, illustrating the conservation and diversification of GAPDH functions throughout plant evolution [23–29].

## Functional roles of GAPDHs in plants

**Figure 2:**
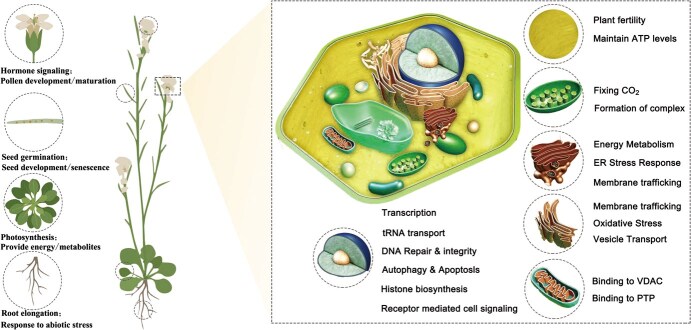
Diverse functional roles of GAPDH across cellular compartments. GAPDH demonstrates extensive multifunctionality, with distinct roles beyond glycolysis and dynamic shifts in subcellular localization. In addition to its pivotal involvement in glycolysis, GAPDH actively participates in regulating gene expression at both transcriptional and post-transcriptional levels, facilitating translation, and mediating vesicular transport. Furthermore, it plays essential roles in receptor-mediated cell signaling, chromatin remodeling, actin and tubulin cytoskeletal organization, DNA repair and integrity maintenance, cellular oxidative stress response, apoptosis, and autophagy regulation. Abbreviations: PTP, permeability transition pore; VDAC, voltage-dependent anion channel.

GAPDH was long considered a classical glycolytic enzyme of minimal significance beyond energy production, often used as a housekeeping gene or protein for internal controls or educational purposes. However, studies have revealed that GAPDH is, in fact, a multifunctional protein with numerous activities unrelated to glycolysis [30–32] and is associated with various stress tolerance in plants [25, 33–37]. Thus, GAPDH represents an archetypal multidimensional protein, whose study is essential for elucidating the links between protein structure and functional diversity, as evidenced by its localization not only in the cytosol but also across the plasma membrane, nuclear membrane, endoplasmic reticulum, Golgi apparatus, and nucleus [3]. Distinct subcellular translocations likely underpin GAPDH’s functional diversity (Fig. 2), and this intricate intracellular ‘fingerprint’ positions GAPDH as a valuable model for exploring how cells regulate the localization of multifunctional proteins to enhance proteomic versatility. Such investigations would reveal the dynamic nature of intracellular protein movement, shedding light on fundamental processes of subcellular transport and the mechanisms by which proteins navigate intracellular pathways to achieve specialized functional diversity.

### Roles of GAPDH in plant growth and development

GAPDH plays several vital roles in plant growth and development beyond its primary function in glycolysis [2, 5]. Although GAPC is integral to fundamental metabolic pathways like glycolysis, its reduced protein levels in transgenic plants typically lead to only mild phenotypic changes under normal growth conditions, suggesting the presence of alternative cytosolic substitutes such as nonphosphorylated GAPN and plastidic GAPDH forms, including A4- and A2B2-GAPDH and GAPCp [2].

Partial suppression of *GAPC* through antisense technology in transgenic *S. tuberosum* has been reported by Hajirezaei et al. (2006). While this manipulation resulted in reduced NAD(H)-dependent GAPDH activity across leaves, stems, and tubers, no significant phenotypic changes were observed [38]. In contrast, studies in *A. thaliana* revealed that knockout (KO) and antisense mutants of *AtGAPC1* exhibited delayed growth, decreased fertility, and alterations in seed and fruit development, which were correlated with a reduced respiratory rate, indicating a metabolic-related phenotype [39] (Table 1). Interestingly, a subsequent study by Guo et al. (2012) demonstrated that *A. thaliana* double mutants lacking both *AtGAPC1* and *AtGAPC2* did not present notable phenotypic alterations under normal conditions [40]; however, these mutants showed increased sensitivity to water stress due to impaired stomatal regulation [41]. This was attributed to the proposed function of AtGAPC1 and AtGAPC2 in sensing hydrogen peroxide (H_2_O_2_) and facilitating the production of phosphatidic acid (PA) in guard cells, a role independent of their enzymatic activity and indicative of moonlighting functions. Additionally, they exhibit roles in gene expression and post-transcriptional regulation, influencing the translatome during light/dark transitions [42]. In a striking contrast, *AtGAPCp* double mutants in *A. thaliana* exhibited severe metabolic-related phenotypes, including dwarfism, arrested root development, and sterility. Despite AtGAPCp contributes minimally to overall glycolytic activity, it plays a critical role alongside phosphoglycerate kinase in supplying 3-phosphoglyceric acid (3-PGA) precursors necessary for serine biosynthesis in plastids [43]. The deficiency of serine in nonphotosynthetic tissues has been identified as the primary cause of inhibited root growth and defects in pollen and embryo development [44]. GAPC-deficient plants show no significant differences in vegetative growth but exhibit a reduction in seed oil content compared to wild-type plants in *A. thaliana* [45]. In *F. ananassa* fruits overexpressing *FaGAPC2*, increased glutamine synthase (GS) activity and decreased glutamate decarboxylase (GAD) activity were observed, suggesting that *FaGAPC2* overexpression reduces citric acid content primarily by inhibiting *FaPEPCK* expression and activating the GS pathway [37]. In addition, *FaGAPC2* is considered a negative regulator involved in the regulation of *F. ananassa* fruit ripening [26]. Reducing the expression of *OsGAPC3* in *O. sativa* can increase the major storage substances (proteins and starch) metabolism, but it also affects the chalkiness area and degree of *O. sativa* [46]. *OsGAPC7* is crucial for energy metabolism and plant development, as its KO mutants exhibit increased starch and soluble sugar content, altered amino acid profiles, and reduced plant height, underscoring its role in carbohydrate metabolism and amino acid homeostasis [47]. PtGAPC1 interacts with PtLTPG14 to regulate glyceraldehyde-3-phosphate (G3P) accumulation and fatty acid (FA) biosynthetic gene expression in poplar [48]. In *S. tuberosum*, *GAPC* genes (*StGAPC1*, *StGAPC2*, and *StGAPC3*) maintain the apical dominance of *S. tuberosum* tubers, with their silencing causing increased axillary bud sprouting without affecting overall plant growth and development [28]. In *Ulmus pumila*, overexpression of *UpGAPDH* suppressed seed germination and caused a dwarf phenotype with reduced leaf numbers compared to wild-type plants at 26 and 41 days of growth [49]. These findings underscore the multifaceted roles of GAPDH in plant metabolism and development, revealing their importance beyond traditional enzymatic functions (Table 1).

**Table 1:** Multifunctional roles of GAPDHs in plants.

**Organism**	**Gene name**	**Localization**	**Biological functions**
***Arabidopsis thaliana***	*GAPC1*	Cytoplasm	Enhancing plant resistance to heat stress [33]
			Affects plant redox metabolism [50]
			Suppresses autophagy [51, 52]
			Delayed growth, reduced fertility, and changes in seed and fruit development [39]
			Plays a role in regulating the translatome during light/dark transitions [42]
			Mediates chloroplast degradation and increases sensitivity to water stress [41]
	*GAPC2*	Cytoplasm	Enhancing plant resistance to heat stress [33]
			Affects plant redox metabolism [50]
			Suppresses autophagy [51]
			Plays a role in regulating the translatome during light/dark transitions [42]
	*GAPCpI*	Plastid	Essential for viable pollen development [44].
	*GAPCpII*	Plastid	Essential for viable pollen development [44]
	*GAPA1*	Chloroplast	Sustains photosynthetic efficiency [11]
			Controlling cell death [53]
	*GAPA2*	Chloroplast	Sustains photosynthetic efficiency [11]
	*GAPB*	Chloroplast	Sustains photosynthetic efficiency [11]
***Oryza sativa***	*GAPC1*	Cytoplasm	A negative regulatory effect on the response to salt stress [54]
	*GAPC3*	Cytoplasm	Enhancing plant tolerance to salt stress [55, 56]
			Affects grain quality traits and improves the nutritional quality [46]
	*GAPC2*	Cytoplasm	A negative regulator of plant autophagy [57]
	*GAPC7*	Cytoplasm	Regulating energy metabolism, especially carbon metabolism [47]
	*GAPA*	Chloroplast	Improves photosynthesis slightly under elevated [CO_2_] conditions [58]
	*GAPB*	Chloroplast	Improves photosynthesis slightly under elevated [CO_2_] conditions [58]
***Triticum aestivum***	*GAPCp1*	Plastid	Enhancing plant resistance to drought stress [59]
	*GAPC1*	Cytoplasm	Increasing the tolerance of plants to drought stress [60, 61]
	*GAPC5*	Cytoplasm	Increasing the tolerance of plants to drought stress [62]
	*GAPDH2*	Cytoplasm	A negative regulator of plant defense [63]
***Nicotiana tabacum***	*GAPC*	Cytoplasm	A negative autophagy regulator [64, 65]
	*GAPCa/GAPCb*	Cytoplasm	Enhance cellular resilience to environmental stress [66]
	*GAPA*	Chloroplast	Recruits a plant virus movement protein to cortical virus replication complexes [67]
***Solanum tuberosum***	*GAPC1/GAPC2/GAPC3*	Cytoplasm	Controlling cold-induced sweetening and apical dominance of tubers [7, 28]
	*GAPC1*	Cytoplasm	Improves nitrogen absorption and utilization [68]
			Inhibits sprout growth in potato tubers [7]
***Paeonia lactiflora***	*GAPC2*	Cytoplasm	Improves thermotolerance [69]
***Ulmus pumila***	*GAPDH*	Cytoplasm	Controlling seed vigor and cell death [49]
			Regulating leaf number and plant growth [49]
***Populus trichocarpa***	*GAPC1*	Cytoplasm	Mediates lipid metabolism and glycolmetabolism [48]
***Fragaria vesca***	*GAPC2*	Cytoplasm	Controlling citric acid content in fruits [37]
***Glycine max***	*GAPDH14*	Cytoplasm	Enhancing plant tolerance to salt stress [70]
***Citrullus lanatus***	*GAPC2*	Nucleus /Cytoplasm	Enhancing plant tolerance to salt stress [24]

### Roles of GAPDHs in plant defense

In mammals, extensive evidence highlights the nonmetabolic functions of GAPDH [59–61, 70, 71], particularly its role as a critical regulator of cell death [53]. S-nitrosylated GAPDH interacts with Siah1, promoting its nuclear translocation and subsequently initiating apoptosis [72]. Furthermore, overexpression of *GAPDH* has been shown to inhibit caspase-independent cell death by enhancing glycolytic activity and autophagy [64, 73]. GAPDH also acts as a prosurvival factor across various cellular processes, including DNA repair, cell cycle progression, and the binding and stability of mRNA [6, 31, 74, 75]. Recently, GAPDHs play a crucial role in plant defense, functioning not only as key enzymes in glycolysis but also in regulating metabolic pathways to provide the substrates necessary for energy and the synthesis of defense-related compounds (Table 1). In *A. thaliana*, GAPDH isoforms AtGAPA, AtGAPCs, and AtGAPCps play key roles in modulating reactive oxygen species (ROS) accumulation and regulating cell death during responses to the bacterial pathogen *Pseudomonas syringae*, which in turn suppresses disease resistance [5]. *AtGAPDH* KO lines displayed enhanced disease resistance when challenged with *P. syringae*, along with accelerated programmed cell death and increased electrolyte leakage during effector-triggered immunity [5]. Overexpression of *AtGAPA1* in protoplasts reduced ROS production and cell death triggered by the apoptosis regulator BAX [53], while the *gapa1–2* and *gapc1* KOs showed constitutive autophagy phenotypes even without nutrient starvation [5]. Conversely, in cassava, MeGAPCs negatively regulate disease resistance against *Xanthomonas axonopodis* pv. *manihotis* (Xam) through interactions with MeATG8b and MeATG8e^25^. Additionally, cytosolic GAPDHs are implicated in viral infection processes, while the chloroplast-localized isoform GAPDH-A facilitates cell-to-cell movement of the Red Clover Necrotic Mosaic Virus by recruiting its movement protein [67]. In *N. tabacum*, NbGAPCs exhibit multifunctional roles in regulating autophagy, the hypersensitive response, and plant innate immunity [65]. The *N. tabacum N* gene, which encodes a TIR-NB-LRR class protein, provides resistance to *Tobacco mosaic virus* (TMV) [60]. The enhanced hypersensitive response (HR) cell death mediated by the *N* gene in GAPC-silenced plants may be attributed to increased autophagy, which is involved in executing immunity-related cell death at the infection site, with excessive autophagy potentially leading to cell death after pathogen exposure [76–78].

Recent studies increasingly confirm that GAPDH also plays an important role in plant responses to environmental stress [34, 79]. Both GAPC isoforms were knocked out in *A. thaliana* double mutants, resulting in higher transpiration water loss under drought stress compared to wild-type plants [40]. Drought stress and abscisic acid (ABA) enhance ROS production in guard cells through NADPH oxidase, and GAPC, a well-known target of H_2_O_2_ in these cells [80], is particularly susceptible to oxidation of its active-site catalytic cysteine residue (Cys152) under oxidative stress [81]. Given the high catalytic reactivity of cysteine residues, prior research has demonstrated that all cytosolic GAPDHs involved in phosphorylation are subject to a range of post-translational modifications in response to ROS [82, 83]. Moreover, AtGAPDH is crucial for regulating stomatal closure in *A. thaliana* in response to water deficit and ABA, acting through its interaction with phospholipase Dδ (PLDδ) to transmit H_2_O_2_ signals [2, 40]. *Arabidopsis thaliana* root tip cells exhibit increased accumulation of both cytosolic and plastidial AtGAPC1 in response to cadmium stress [17, 84]. Additionally, AtGAPDH expression rises in *A. thaliana* under conditions of water scarcity [85]. *AtGAPC* overexpression boosts heat tolerance in seedlings and promotes the expression of heat-inducible genes, while *GAPC* KO leads to reduced heat tolerance and downregulation of these genes in *A. thaliana* [33]. The expression levels of the *ObGAPDH* gene in different cultivars of *Ocimum basilicum* exhibit variation under drought-induced stress conditions [34]. Previous studies have shown that H_2_O_2_ inhibits the enzymatic activity of recombinant GAPC1 and GAPA1 proteins [86]. In salt-tolerant *O. sativa* lines, there is notable overexpression of a cytosolic OsGAPC3 isoform. Moreover, GAPDH has been found to form immune complexes with protein kinases activated during osmotic stress in salt-treated *N. tabacum* cells [66].

## The regulation mechanism of GPADHs in plants

**Figure 3:**
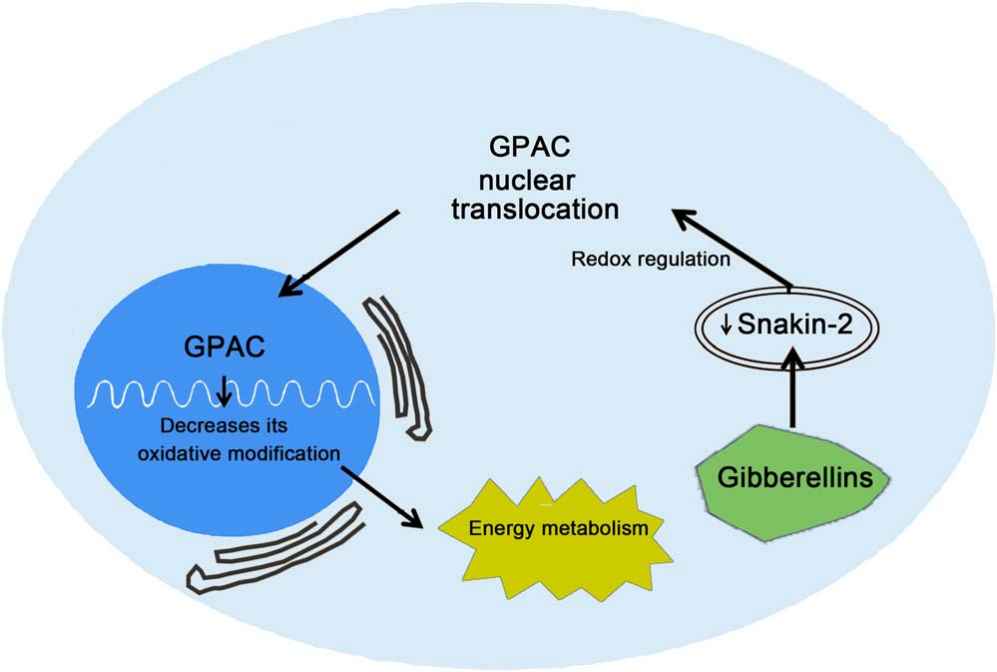
A novel nuclear function of GAPDH in plants. Gibberellins (GA) suppresses Snakin-2 expression, and the subsequent interaction between Snakin-2 and GAPC enhances GAPDH activity, thereby altering energy metabolism; additionally, this interaction diminishes oxidative modification of GAPC and facilitates its nuclear accumulation.

Interestingly, while GAPDH possesses a nuclear export signal, it does not have a nuclear localization signal (NLS) [87], suggesting that other mechanisms are involved in its translocation to the nucleus. The functional versatility of GAPDH is largely due to its sensitivity to reversible redox post-translational modifications (RPTM) at the catalytic cysteine and to protein–protein interactions that influence its localization between the cytosol and the nucleus. For GAPDH, translocation from the cytosol to noncytosolic compartments leads to the acquisition of distinct functions beyond its traditional glycolytic role [3]. An example of constitutive regulation is illustrated in Fig. 3, highlighting a novel nuclear function of GAPDH: inhibition of *S. tuberosum* tuber sprout growth through interaction with Snakin-2 [7]. Snakin-2, a plant-specific member of the Snakin/GASA family, plays a key role in regulating growth and responding to various stress conditions [88, 89]. In this regard, the interaction between GAPC and Snakin-2 enhances GAPC1 activity, impacting energy metabolism and delaying tuber germination [7]. Additionally, Snakin-2 reduces oxidative modification of GAPC and promotes its nuclear accumulation. Nuclear-localized GAPC may interact with transcription factors or gene promoters, thereby modulating the expression of key genes involved in tuber germination [7]. The data implicate GAPDH in various cellular processes, indicating that it translocates to the nucleus, where it interacts with transcription factors or gene promoters, ultimately promoting plant growth and development [90, 91]. This raises critical questions about how identical GAPDH molecules acquire diverse subcellular localizations and functions. Possible mechanisms include specific targeting motifs or post-translational modifications, likely involving carrier proteins for precise transport. Given GAPDH’s highly conserved sequence, the selective recruitment of identical molecules to various cellular compartments, such as polysomes or the nucleus, remains an open question that requires further investigation.

Current data indicate that post-translational modifications of GAPDH are extensive and varied, encompassing phosphorylation at serine residues [92], tyrosine [9,2], and the active-site cysteine-149 [72, 93]; pyruvylation by 3-bromopyruvate [94]; acetylation at lysines 160 [95], 117, 227, 251 [96], 1,21, and 231 [33]; poly ADP-ribosylation induced by hyperglycemic stress [97]; and O-linked N-acetylglucosamine glycosylation at threonine-227 [98]. Furthermore, a recent study has investigated the functional implications of these modifications. Differential post-translational modifications likely provide a mechanism for the constitutive subcellular regulation of multifunctional proteins, including GAPDH [2, 33, 99]. Analyzing GAPDH complexes with RNA, DNA, or proteins, such as mRNA, telomeric DNA, and associations with individual proteins or multiprotein complexes, will facilitate experimental testing of this theory. Each complex is presumed to contain a GAPDH variant characterized by a unique post-translational modification. Similar investigative methods can be applied to other multidimensional proteins.

**Figure 4:**
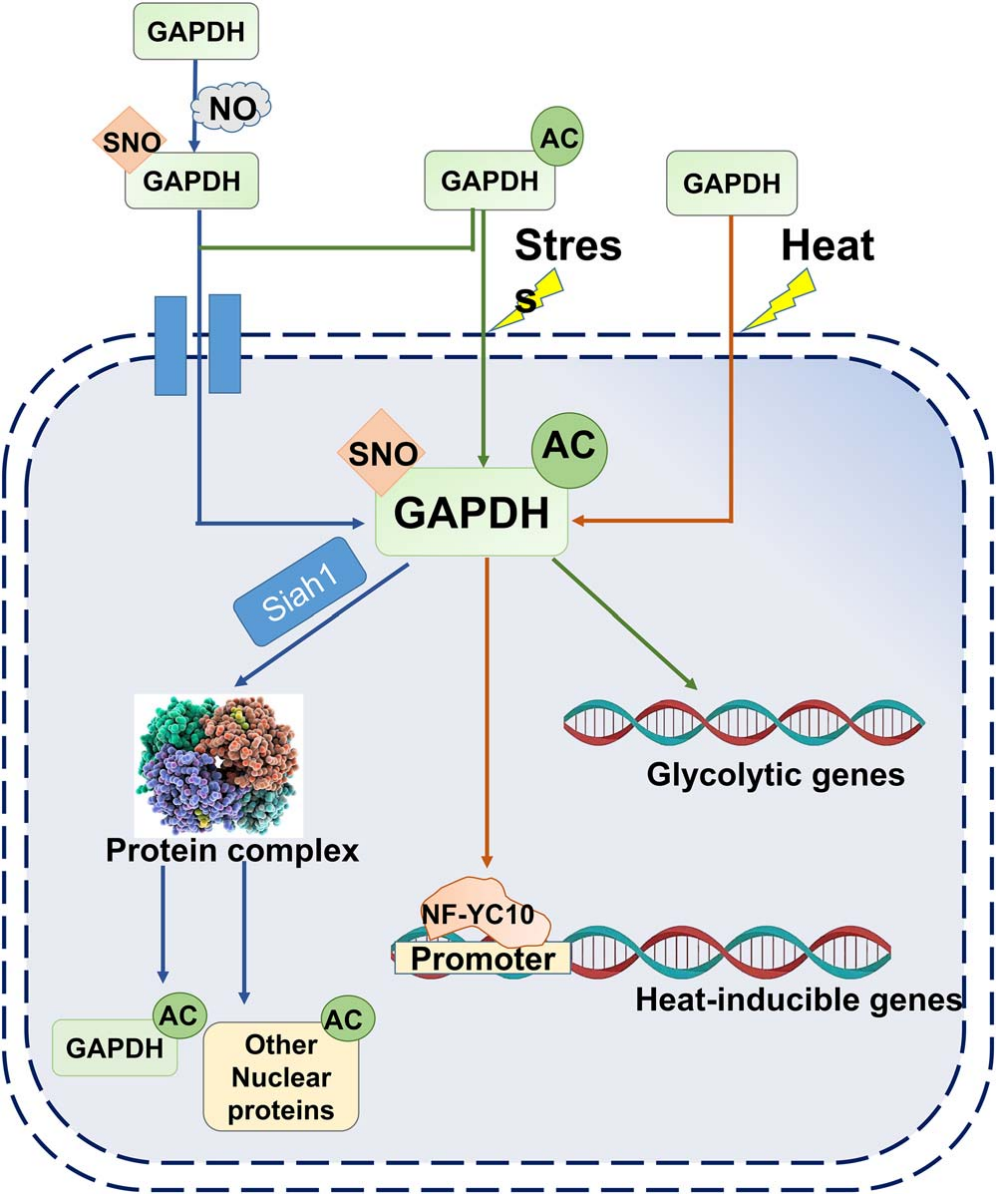
GAPC that normally exists in the cytosol enters the nucleus in response to heat and various other stress conditions. Under stress conditions, GAPC undergoes post-translational modifications, such as acetylation, which facilitates its translocation into the nucleus through vesicle trafficking. Within the nucleus, GAPC interacts with the transcription factor NF-YC10 to enhance the expression of heat-inducible genes, thereby increasing *A. thaliana* tolerance to heat stress.

One of the mechanisms by which GAPC participates in stress responses is its stress-induced nuclear translocation. In *A. thaliana*, a small pool of AtGAPC accumulates in the nucleus in response to cadmium, bacterial flagellin, phosphatidic acid, heat stress, and hydrogen sulfide treatments [5, 33, 84, 100–102]. Recent findings indicate that distinct pathways govern GAPDH’s intracellular transport for each specific function. During the stress response of *A. thaliana*, AtGAPC1 transduces H_2_O_2_ signals by interacting with PLDδ [40]. Similarly, cadmium and other oxidation-inducing substances stimulate the nuclear accumulation of GAPC1 in root tip cells [84]. AtGAPC1 negatively regulates CHLOROPLAST VESICULATION (CV)-mediated chloroplast degradation and stress responses by suppressing CV-clathrin heavy chain2 (CHC2) interaction and clathrin-assisted CVV budding under water stress [41]. In response to heat stress, AtGAPC binds to AtNF-YC10, and this interaction facilitates the complex’s transport into the nucleus, driven by the NLS of NF-YC10 [33] (Fig. 4). The interaction between AtGAPC and NF-YC10 likely influences the structural stability and regulatory function of the transcriptional complex, thereby modulating the expression of heat-inducible genes. However, AtGAPC does not directly bind key components of the complex, such as *DREB2A*, nor does it significantly impact NF-YC10 interactions with individual binding partners. It is possible that the role of AtGAPC involves post-translational modifications or nuclear factors yet to be identified, which may influence the function of the NF-Y heterocomplex. Overexpression of *AtGAPC* enhances the expression of both *DREB2A*-regulated genes and other heat-inducible genes not directly associated with *AtDREB2A*, including *AtHsfA2*. The promoter of *AtHsfA2* is directly bound by AtNF-YC10 during heat stress, and its expression is increased by AtNF-YC10 overexpression, indicating a regulatory pathway independent of *AtDREB2A*. These findings suggest that AtGAPC modulates the ability of AtNF-YC10 to bind target promoters, such as *AtHsfA2*, thereby regulating heat-inducible gene expression through alternative mechanisms (Fig. 4). In *N. tabacum*, NbGAPC1 and NbGAPC2 interact with chaperones like NbATG3 to suppress ATG3-induced autophagy, with these interactions dynamically regulated by external factors such as ROS that modulate GAPDH activity and its affinity for chaperones [65]. Additionally, silencing *NbGAPCs* enhances *N* gene-mediated cell death and resistance to incompatible pathogens (*Tobacco mosaic virus* and *P. syringae* pv *tomato* DC3000) as well as the compatible pathogen *P. syringae* pv *tabaci* [65]. In *S. tuberosum*, StGAPC1, StGAPC2, and StGAPC3 are constitutively expressed and cold-inducible in tubers, and their silencing reduces GAPDH activity, leading to increased reducing sugar accumulation in cold-stored tubers and loss of apical dominance due to cell death in the tuber apical bud meristem mediated by interactions with the autophagy-related protein StATG3 [28]. This bidirectional regulation underscores the multifaceted roles of GAPCs in autophagy, hypersensitive response, and plant innate immunity, while also explaining the phenotypic changes observed when GAPDH isoforms are silenced or overexpressed.

Additionally, post-translational modifications are believed to play a pivotal role in initiating the stress-dependent nuclear translocation of GAPC. Under specific stress conditions, the reactive catalytic cysteine of GAPC is subject to thiol modifications, including S-nitrosylation, S-sulfhydration, and S-glutathionylation [38, 102]. S-nitrosylation plays a critical role in regulating GAPC activity, as its levels transiently increase in salt-treated *N. tabacum* BY-2 cells [66]. Mutation of GAPC’s active cysteine residues to serine disrupts its salt-induced interaction with osmotic stress-activated protein kinase (OSAK) in the nucleus, while leaving their cytoplasmic interaction unaffected. This highlights the importance of S-nitrosylation in mediating GAPC’s nuclear function under salt stress [66]. Certain lysine residues may undergo acetylation, which facilitates nuclear translocation [56]. Salt stress induces acetylation of OsGAPC1 at four critical sites (K120, K196, K253, and K265), which is essential for its nuclear export. Mutating these sites to alanine abolishes acetylation and disrupts the response to salt stress [54]. Furthermore, lysine ubiquitination by an E3 ubiquitin ligase has been identified as a potential mechanism for GAPC’s localization to the nucleus [103]. Recent studies have revealed that the E3 ubiquitin-protein ligase SINAL7 directly interacts with GAPC1 and regulates its nuclear abundance, with K231 ubiquitination playing a key role [103, 104]. Mutation of K231 disrupts this interaction, and SINAL7 overexpression enhances drought tolerance by upregulating *DREB2A* and reducing stomatal aperture [103, 104]. These findings suggest that ubiquitination by SINAL7 influences heat-dependent nuclear translocation of GAPC [103].

## Conclusion and future perspective

GAPDH, long considered a ‘housekeeping’ gene restricted to glycolysis, has gained increasing attention for its unexpected roles in diverse cellular processes. While its high abundance initially led to its dismissal as an artifact in nonglycolytic contexts, extensive research has uncovered its complex physiological significance [74]. GAPDH functions across all major cellular compartments, playing key roles in redox signaling, autophagy, immunity, and transcriptional regulation. Although significant progress has been made in studying these functions in several model plants, the specific roles of GAPDHs in horticultural crops remain largely unexplored. Addressing these gaps could uncover unique mechanisms by which GAPDH contributes to stress resilience and developmental regulation in horticultural plants.

Many of the nonglycolytic functions described earlier depend on the nuclear translocation of GAPDH. Lacking an NLS but containing a nuclear export signal [105], GAPDH relies on chaperone interactions (e.g. ATG3, SGL, or NF-YC10) and post-translational modifications like S-nitrosylation and acetylation to enter the nucleus and regulate gene transcription [33, 54, 65]. In horticultural crops, such mechanisms may be critical for GAPDH’s involvement in stress responses and developmental transitions, such as fruit ripening and tuber dormancy. For example, nuclear GAPDH is enriched in the chromatin of several glycolytic genes to activate target gene expression, although it does not function as a typical sequence-specific DNA-binding transcription factor [56]. Investigating how these mechanisms operate in crops like *Malus domestica*, *Prunus persica*, or *S. tuberosum* could reveal novel pathways for regulating key traits under both normal and stress conditions.

The multifunctionality of GAPDH offers great potential for improving productivity and resilience of horticultural crops. Its roles in redox regulation and post-translational modification suggest it could be manipulated to enhance abiotic stress tolerance, such as chilling resistance in fruits or oxidative damage prevention in leafy greens during storage. Moreover, the effects of GAPDH on developmental processes like dormancy and carbohydrate partitioning, as demonstrated in *S. tuberosum* [28, 35], highlights opportunities for improving yield and quality in crops with similar traits, such as onions and garlic. By employing advanced genome-editing tools like CRISPR/Cas9, researchers could fine-tune GAPDH isoforms to optimize stress responses, sugar metabolism, and developmental timing. Uncovering autophagy-related proteins and transcriptional regulators that interact with GAPDH could further unlock pathways for tailored crop improvement. In summary, as the multifunctionality of GAPDH continues to be uncovered, it holds great promise not only as a target for improving crop performance but also as a model for investigating the evolutionary adaptations of plant metabolic enzymes.

## Data Availability

All relevant data are included in the manuscript.
